# Identification of QTLs and possible candidate genes conferring sheath blight resistance in rice (*Oryza sativa* L.)

**DOI:** 10.1186/s40064-015-0954-2

**Published:** 2015-04-11

**Authors:** Shailesh Yadav, Ghanta Anuradha, Ravi Ranjan Kumar, Lakshminaryana Reddy Vemireddy, Ravuru Sudhakar, Krishnaveni Donempudi, Durgarani Venkata, Farzana Jabeen, Yamini Kalinati Narasimhan, Balram Marathi, Ebrahimali Abubacker Siddiq

**Affiliations:** Institute of Biotechnology, Acharya N G Ranga Agricultural University, Hyderabad, 500030 India; Seed Research and Technology Centre, Rajendranagar, Hyderabad, 500030 India; Divison of Plant Pathology, Directorate of Rice Research, Rajendranagar, Hyderabad, 500030 India; International Rice Research Institute, DAPO Box 7777, Metro Manila, Philippines; Department of Molecular Biology and Genetic Engineering, BAU, Sabour, 813210 India

**Keywords:** Rice, Sheath blight, QTL mapping, Bulk segregant analysis (BSA), Defense response genes

## Abstract

Sheath blight, caused by the pathogenic fungus *Rhizoctonia solani* Kühn, is one of the most devastating diseases in rice. Breeders have always faced challenges in acquiring reliable and absolute resistance to this disease in existing rice germplasm. In this context, 40 rice germplasm including eight wild, four landraces, twenty- six cultivated and two advanced breeding lines were screened utilizing the colonized bits of typha. Except Tetep and ARC10531 which expressed moderate level of resistance to the disease, none could be found to be authentically resistant. In order to map the quantitative trait loci (QTLs) governing the sheath blight resistance, two mapping populations (F_2_ and BC_1_F_2_) were developed from the cross BPT-5204/ARC10531. Utilizing composite interval mapping analysis, 9 QTLs mapped to five different chromosomes were identified with phenotypic variance ranging from 8.40 to 21.76%. Two SSR markers namely RM336 and RM205 were found to be closely associated with the major QTLs *qshb7.3* and *qshb9.2* respectively and were attested as well in BC_1_F_2_ population by bulk segregant analysis approach. A hypothetical β 1–3 glucanase with other 31 candidate genes were identified in silico utilizing rice database RAP-DB within the identified QTL region *qshb9.2*. A detailed insight into these candidate genes will facilitate at molecular level the intricate nature of sheath blight, a step forward towards functional genomics.

**Electronic supplementary material:**

The online version of this article (doi:10.1186/s40064-015-0954-2) contains supplementary material, which is available to authorized users.

Sheath blight, caused by *Rhizoctonia solani* Kühn of the anastomosis group AG1- IA, is potentially a devastating disease of rice (*Oryza sativa*) in the temperate and tropical rice growing regions of the world (Teng et al. 1990). In recent years, the disease has assumed serious epiphytotic proportions causing earnest crop losses under intensive rice production systems characterized by abundant application of nitrogenous fertilizers, high planting density, and extensive adoption of high-yielding cultivars (Slaton et al. 2002).

Breeding for sheath blight (ShB) resistance has been a futile exercise so far, mainly because of lack of reliable stable sources of resistance in rice germplasm (Jia et al. 2009; Zuo et al. 2010; Srinivasachary and Savary 2011; Liu et al. 2013). Though rice literature is replete with reports on the occurrence of sources with varied levels of resistance have been found as in Teqing (Li et al. 1995; Pinson et al. 2005), Jasmine 85 (Pan et al. 1999; Zou et al. 2000; Liu et al*.*^2009^), Minghui63 (Han et al. 2002), Xiangzaoxian 19 (Che et al. 2003), WSS2 (Sato et al. 2004), Pecos (Sharma et al. 2009) and Tetep (Sha and Zhu, 1989; Channamallikarjuna et al. 2010) and wild rices *O. rufipogon*, *O. nivara* etc. (Ram et al. 2008; Eizenga et al. 2013). Intensive study of the resistance in such sources reveals it to be quantitative in nature controlled possibly by polygenes (Sha and Zhu 1989; Li et al. 1995; Pinson et al. 2005; Zuo et al. 2013 It is widely believed that quantitative nature of resistance could be the expedient for evolving varieties with durable/horizontal resistance (Young, 1996; Poland et al. 2009). Precise mechanism underlying the quantitative resistance as yet not well understood, could be attributed to the host plant defense system through PR proteins in response to attack by pathogen (Datta et al. 1999; Vanloon and Strien 1999). Over-expression of PR proteins, including *chitinases* (PR-3*), β-1, 3-glucanases* (PR-2), *thaumatin* like proteins (PR-5), and other plant or microbe derived antifungal proteins in transgenic plants is known to provide resistance to sheath blight (Lin et al. 1995 and Datta et al. 2001).

With the rapid development of molecular marker technology, there have been significant advances in mapping ShB resistance QTLs: To date, around 50 ShB resistance quantitative trait loci (ShBR QTLs) have been mapped to all the 12 rice chromosomes (Jia et al*.*^2009^; Zuo et al. 2010; Xu et al. 2011 and Wang et al. 2012). Several recent studies have explore the possible candidate genes *viz. Chitinase, Glucanase, Glutathione S-Transferase* and *Kinase* protein within the mapped QTL region may responsible for sheath blight resistance (Channamallikarjuna et al. 2010; Silva et al. 2012; Zuo et al. 2014). Though most of the ShBR QTLs identified so far are of only limited effects on ShB resistance, instances of some showing expected effect were not uncommon. For instance Zuo et al. (2007) reported introgression of the QTL, *qSB-11*^*LE*^ and observed reduced grain loss by 10.71% in Lemont background under severe disease infestation in field trials. Pinson et al*.* (2005) predicted that *qSB-9*^*TQ*^ and *qSB-3*^*TQ*^ could possibly reduce the crop loss due to ShB by 15% when introduced into Lemont. Yin et al. (2010) and Wang et al. (2012) have found that pyramiding of diverse ShBR QTLs could help achieve higher levels of resistance to ShB. Thus, it is likely that the level of resistance to the disease could be raised by pyramiding diverse ShBR QTLs differing in their level of moderate resistance.

Keeping in view this possibility the present investigation was conducted to i) identify ShB resistance source(s), ii) establish their genetic diversity by molecular mapping of the QTLs associated with sheath blight resistance by using F_2:3_, iii) validation of markers associated with sheath blight resistance in BC_1_F_2,_ iv) to identify the possible candidate genes in the QTL region by *in silico* analysis.

## Materials and methods

### Plant material

Forty rice germplasm including improved cultivars (26), wild (8), landraces (4) and advanced breeding lines (2) were screened to identify resistance sources for sheath blight disease. Of these, some genotypes were previously reported to be sources of resistance to the disease (Table 1).

**Table 1:** **List of rice germplasm screened for Sheath Blight Resistance**

**Sr. no.**	**Genotype**	**Source**
**Wild Accessions**		
**1**	*Oryza rufipogon* AC100488	DRR-Hyderabad
**2**	*O. rufipogon* AC 100368	DRR-Hyderabad
**3**	*O. rufipogon* AC 100490	DRR-Hyderabad
**4**	*O. rufipogon* AC100483	DRR-Hyderabad
**5**	*O. nivara* AC100456	DRR-Hyderabad
**6**	*O. nivara* AC100396	DRR-Hyderabad
**7**	O. *nivara* AC 100395	DRR-Hyderabad
**8**	O. *nivara* AC 100110	DRR-Hyderabad
**Landraces**		
**9**	N-22	NDUAT-Faizabad
**10**	Tetep	NRCPB- New Delhi
**11**	Moroberakan	DRR-Hyderabad
**12**	ARC 10531	AAU-Assam
**Cultivated**		
**13**	Swarna	ANGRAU-Hyderabad
**14**	Rajeswari	OUAT-Odisha
**15**	Swarnadhan	DRR-Hyderabad
**16**	Kavya	ANGRAU-Hyderabad
**17**	IR-64	IRRI,Philippines
**18**	Lalnakandha	TNAU-Coimbatore
**19**	Naveen	CRRI-Cuttack
**20**	MTU1061	ANGRAU-Hyderabad
**21**	Vandana	ANGRAU-Hyderabad
**22**	Pusa basmati	IARI-New Delhi
**23**	MTU1001	ANGRAU-Hyderabad
**24**	Sonasali	DRR-Hyderabad
**25**	BPT-5204	ANGRAU-Hyderabad
**26**	Jaya	DRR-Hyderabad
**27**	TKM-6	TNAU-Coimbatore
**28**	Nilagiri	OUAT-Odisha
**29**	Jyothi	KAU-Kerala
**30**	WGL-32100	ANGRAU-Hyderabad
**31**	Ghanteswari	ANGRAU-Hyderabad
**32**	MTU-1010	ANGRAU-Hyderabad
**33**	WGL-14	ANGRAU-Hyderabad
**34**	Chandan	CRRI-Cuttack
**35**	Surekha	ANGRAU-Hyderabad
**36**	Khandagiri	OUAT-Odisha
**37**	JGL-3844	ANGRAU-Hyderabad
**38**	Jaganath	TNAU-Coimbatore
**Advanced Breeding Lines**		
**39**	RIL-45	NRCPB- New Delhi
**40**	RIL-140	NRCPB- New Delhi

### Evaluation of rice germplasm for sheath blight resistance

The screening for the disease reaction was carried out in Kharif 2011 at the Institute of Biotechnology, ANGRAU under the artificial epiphytotic conditions in a hot humid chamber. The material was sown in 3 replications in separate pots and maintained in the humid chamber at optimum humidity (90%) and temperature (28-30°C), which are very favorable for the disease development.

### Multiplication of the pathogen

In the present study, the most virulent local Rajendranagar isolate of rice sheath blight pathogen *R. solani* AG 1-IA (Wamishe et al. 2007) obtained from the Division of Plant Pathology, Directorate of Rice Research, Hyderabad and utilized used for disease screening.

Before the inoculation, the fungus was cultured in potato dextrose agar (PDA) medium at 28°C for 3–4 days, followed by transferring of disc of medium with mycelia for multiplication. The inoculum of the virulent isolate was multiplied by following the procedure described by Bhaktavatsalam et al. (1978). Typha stem bits of 4–5 cm long were washed exhaustively and soaked in typha medium for 5 minutes. Subsequently, they were filled loosely to 1/3 volume of 500 ml conical flask and autoclaved for 20 minutes each for two consecutive days. The sterilized flasks with typha were inoculated with 5 mm diameter disk of actively growing mycelium of *R. solani* AG1-IA and incubated for one week at 28 ± 2°C. Then colonized typha stem bits so developed were utilized as inoculum.

### Pathogen inoculation and disease scoring

Plants at maximum tillering stage were inoculated with *R. solani* by placing the typha pieces infected with *R. solani* between tillers in the central region of rice hills 5–10 cm above waterline. So inoculated plants were then kept in a humid chamber made of clear plastic maintained at 28°C under 14-hr day light for 2 weeks to allow disease development in the greenhouse. Humidity was maintained between 80 and 90% from the time of inoculation for disease evaluation. Inoculated plants were scored for disease reaction as percentage relative lesion height (RLH%) 14 days after inoculation as under:

$$ \mathrm{R}\mathrm{L}\mathrm{H}\%\kern0.5em =\kern0.5em \frac{\mathrm{Lesion}\ \mathrm{height}\ \left(\mathrm{cm}\right)}{\mathrm{Plant}\ \mathrm{height}\kern0.75em \left(\mathrm{cm}\right)}\kern0.5em \times 100 $$

The scoring was done on 0–9 scale of Standard Evaluation System (SES) for rice (IRRI, 2002) detailed in Additional file 1: Table S1.

### Experimental population

In the present study, ARC10531 and Tetep were identified as moderately resistant to sheath blight among the 40 rice germplasm screened. Tetep is a known source for moderate resistance to the disease and QTLs relating to resistance has already been reported by Channamallikarjuna et al. (2010). Hence, the present study was confined to ARC 10531 for development of mapping population and mapping of QTLs conferring resistance.

One mapping population consisted of 210 F_2:3_ progeny lines derived from the cross between the susceptible BPT-5204 and moderately resistant ARC10531. The susceptible parent BPT-5204 is the late -maturing, fine grain, semi-dwarf and high yielding variety but highly susceptible to *R. solani.* The resistant parent ARC 10531 is a late-maturing, bold grain and tall land race from Assam with moderate resistance to ShB. The other mapping population (BC_1_F_2_) comprising 150 plants derived from the same cross (BPT-5204/ARC10531) planted in the green house at IBT (ANGRAU) were used to validate the markers linked to sheath blight resistance QTLs.

### Phenotyping of F_2:3_ and BC_1_F_2_ population

The 210 plants of F_2:3_ and 150 of BC_1_F_2_ population derived from the cross (BPT-5204/ARC10531) were grown in pots as well as under soil bed conditions. The plants were inoculated with *R. solani* multiplied in typha bits and disease reaction for the disease was recorded as per the Standard Evaluation System described earlier.

### Bulk segregant analysis

Bulk Segregant Analysis (BSA) is as an efficient strategy for identifying the DNA markers linked to the gene(s) or genomic regions of interest (Michelmore et al. 1991). By making DNA bulks, all loci are randomized, except for the region containing the gene of interest. In this approach, the markers are screened across the parents and two bulks. Polymorphic markers may represent markers that are linked to the gene or QTL of interest (Collard et al. 2005). DNA bulks of plants showing resistant reaction and of those found susceptible were prepared from BC_1_F_2_ population. This was done by pooling aliquots, containing equivalent amounts of DNA approximately, 50 ng/μl from each of ten highly resistant and ten highly susceptible plants of the BC_1_F_2_ generation predicated on phenotypic observations. Seventy polymorphic SSR primers were utilized for screening the parents and the two bulk DNA samples.

### Construction of molecular linkage map

The total genomic DNA was extracted of young leaves from each of the F_2_ plants along with those of the two parental lines - BPT5204 and ARC10531as per the method described by Murray and Thompson (1980). The concentration of the DNA was detected with the aid of ultraviolet spectrophotometer. The DNA quality was judged by running on 0.8% agarose gel electrophoresis stained with ethidium bromide (EtBr) (10 mg/ml) using 1× TAE buffer at constant voltage 70 V for 30 minutes and visualized under UV light. The DNA was amplified through PCR using reaction mixture (total volume 10 μl), containing 20 ng template DNA, 10 mM Tris–HCl, 50 mM KCl, 2.5 mM MgCl_2_, 10 mM dNTPs, 5 pmol each primers and 1.0 U Taq DNA polymerase. The PCR reaction was performed as DNA denaturation at 94°C for 4 minutes followed by 35 cycles (94°C for 1 minutes, 55°C for 1 minutes, 72°C for 1 minutes) and final extension step (at 72°C for 10 minutes). The PCR product was subjected to electrophoresis on 4% metaphor agarose. The DNA fragments were then visualized under UV-transilluminator and documented using a gel documentation system (BIO-RAD Gel Doc™ XR, USA).

Identification of sufficient number of markers revealing polymorphism among the parental lines is a prerequisite for the construction of a genetic linkage map. In this study, parental polymorphism survey was carried out between the two parents (ARC10531 and BPT-5204) using a total of 500 SSR markers spanning all the 12 chromosomes of rice (http://www.gramene.org/). The list of polymorphic markers with their details is depicted in Additional file 1: Table S2.

Seventy polymorphic markers were used for genotyping of the 210 F_2_ individuals along with parental lines. A linkage map was constructed by using MAPMAKER 3.0 (Cambridge, MA, USA; Lander et al. 1987). The map distances were calculated based on Kosambi’s function (Kosambi, 1944).

### QTL mapping

Identification of QTLs for ShB resistance was completed using the software QTL cartographer v. 2.5 (Wang et al. 2004http://statgen.ncsu.edu/qtlcart/WQTLCart.htm). To identify additional QTLs that may have been masked by the major QTLs, composite interval mapping (CIM) method was employed. Genome-wide threshold values (α = 0.05) were used to detect putative QTLs on the basis of the results of 1000 permutations (Churchill and Doerge 1994). The likelihood-ratio (LR) test statistic used was -2ln (L0/L1), where L0/L1 is the ratio of the likelihood under the null hypothesis (there is no QTL in the interval) and the alternative hypothesis (there is a QTL in the interval). The presence of a putative QTL was declared if the LOD threshold was larger than 3.0 (Basten et al. 2002). The QTLs were deemed to exist only at positions where an LOD score exceeded the corresponding significant threshold. Estimation of the position, genetic effects, and percentage of phenotypic variation of the QTLs were done at the significant LOD peak in the region under consideration. The proportion of phenotypic variation explained by each QTL was calculated as R^2^ value, and the degree of dominance of a QTL was estimated as the ratio of dominance effect to additive effect.

## Results and discussion

### Screening of rice germplasm for sheath blight resistance

**Figure 1:**
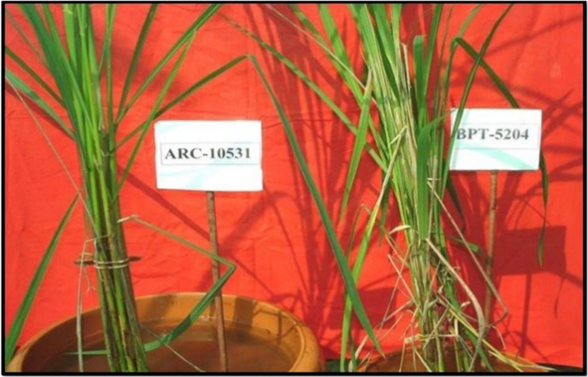
Disease reaction of ARC 10531 and BPT-5204 (observations were recorded after 14 days of inoculation).

**Figure 2:**
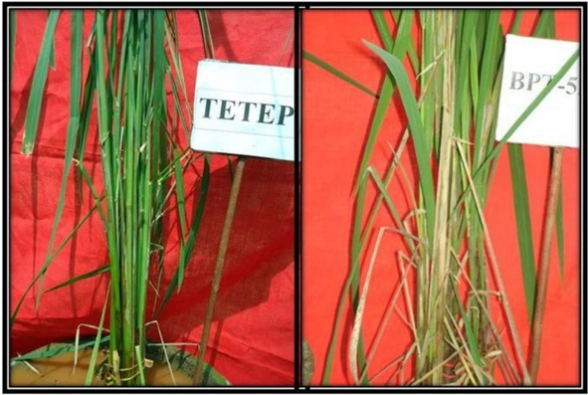
Disease reaction of Tetep and BPT-5204 (observations were recorded after 14 days of inoculation).

Among the 40 rice germplasm comprising wild accessions, land race, improved cultivars and advanced breeding lines (RIL-45 and RIL140) screened for sheath blight resistance, only two genotypes viz Tetep and ARC10531 found to in the range of 21-30% denoting them to be of moderate resistance (Table 2). Tetep is a well reported source of resistance to sheath blight and several QTLs have already been mapped by number of researchers. The land race ARC 10531 identified in the present study was observed with equal levels of resistance as that of Tetep, could be used as an alternate source for ShB resistance (Figures 1 and 2). The information generated in the present study would be valuable in future rice breeding programmes aimed at improving resistance to sheath blight.

**Table 2:** **Grouping of rice genotypes based on their reactions against the fungus** ***R. solani,*** **AG1-IA**

**Grade**	**Relative lesion height (%)**	**Reaction**	**Genotypes**
**0**	0	Immune	Nil
**1**	1-20	Resistant	Nil
**3**	21-30	Moderately resistant	Tetep, ARC-10531
**5**	31-45	Moderately susceptible	RIL-140 of cross HP2216× Tetep, Swarnadhan, MTU 1061, Kavya, Naveen, *Oryza rufipogon* AC 100490,
**7**	46-65	Susceptible	Rajeswari, Moroberakan, Vandana, Lalnakandha, MTU 1001, *Oryza nivara* AC100395*,* AC100396*, Oryza rufipogon* AC100488, Sonasali and Pusa Basmati,
**9**	66-100	Highly susceptible	TKM-6, Nilagiri, Jyothi, WGL32100, Chandan,N-22,Ghanteswari, MTU-1010, Jaya, Jaganath, WGL14, Surekha, JGL-3844,IR-64, Khandagiri, *Oryza nivara* AC100456, AC100110*Oryza rufipogon* AC100483 and AC100368, RIL-45 of cross HP2216x Tetep, BPT-5204,

Several researchers have attempted in the past to identify sources of resistance to ShB by screening a large number of wild species, landraces, local and improved cultivars, advanced breeding lines using different screening techniques (Srinivasachary and Savary 2011). They broadly included use of colonized typha bits (Bhaktavatsalam et al. 1978), a toothpicks infected with *R. solani* (Zou et al*.*^2000^), broadcasting of inoculum on rice plants (Li et al. 1995; Savary et al. 1995; Singha and Borah 2000 and Han et al. 2002), infected rice grain–hull mixtures (Pan et al. 1999; Willocquet et al. 2000), detached leaf technique (Prasad and Eizenga, 2008) and micro-chamber method (Jia et al. 2009) under controlled greenhouse conditions. In comparison with field ShB evaluation, the micro-chamber and mist-chamber assays were simple, precise and more reliable assays methods in tagging ShB resistance (Liu et al. 2009; Jia et al. 2009). In the present study controlled chamber method was adopted by keeping the potted plants inoculated with typha bits under greenhouse condition, maintained at optimum humidity (90%) and temperature (28-30°C) for disease development.

As for disease development and expression, earlier reports suggest plant height to be related to disease severity (Sharma et al. 2009; Liu et al*.*^2014^) reveal relatively tall statured plants favor disease escape delaying the spread of the pathogen to canopy. In the present study, ARC 10531 and Tetep exhibited low RLH% possibly due to a well-developed mechanism against sheath blight disease as well as their tall stature nature which delays the spread of the pathogen to the canopy in these moderately resistant line. The statement cannot be generalized as in the present study other varieties and wild accessions tall in stature expressed susceptibility towards ShB disease. Pace of disease development need not be due to plant height alone. Possibly there may be other factors associated with the resistance gene warranting more detailed study of such an association. Channamallikarjuna et al. 2010 similarly viewed that sheath blight resistance quantified in Tetep might be due to the molecular mechanisms involved in host- pathogen interaction and not due to any morphological adaptation to evade disease. The heading date is also associated significantly with ShB resistance and the varieties with later maturing are generally more resistant to ShB (Zuo et al. 2010; Srinivasachary and Savary 2011).

The present study though could not succeed in finding a strong source of resistance to sheath blight, assumes importance in identifying additional source of moderate resistance to the diseases in ARC10531 besides the already known source Tetep. If they are found to be of diverse genetics, they would prove a base for pyramiding them and expect enhanced level of resistance.

### Phenotype distribution analysis

**Figure 3:**
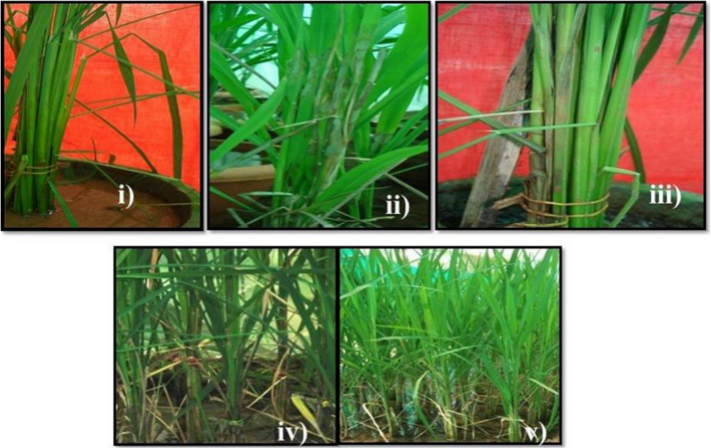
Disease reaction of **i)** ARC-10531, **ii)** BPT-5204, **iii)** F_1,_ **iv** and **v)** individuals of mapping population F_2:3_ showing moderately resistant and susceptible reaction.

**Figure 4:**
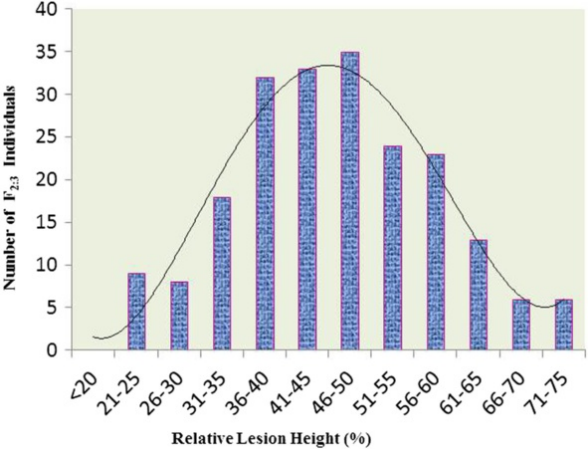
Frequency distribution curve of Sheath Blight disease incidence among 210 F_2:3_ individuals derived from the cross (BPT-5204 × ARC10531).

**Figure 5:**
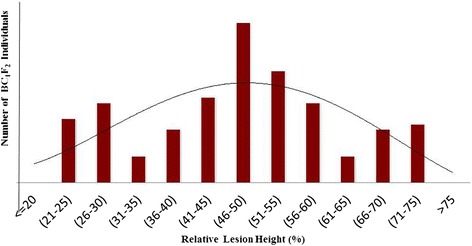
Frequency distribution curve of sheath blight disease incidence among 150 individuals of BC_1_F_2_ population derived from the cross (BPT-5204 × ARC10531).

The F_2:3_ progenies exhibited significant phenotypic variance for the disease reaction (Figure 3). Frequency distribution of F_2:3_ was continuous and fitted into normal distribution as expected from a quantitative trait (Figure 4). The population appeared to be skewed towards the susceptible range of 40-50% and the percentage relative lesion height ranging between 21% and 75% with the mean value of 31.51%. Similar to the F_2:3_ population, the frequency distribution of the BC_1_F_2_ population was also skewed towards susceptibility (Figure 5). Broadly the findings were in agreement with those of Sharma et al. (2009), who also observed the frequency distribution to be continuous showing skewness towards susceptibility.

### Linkage map construction and QTL analysis

**Figure 6:**
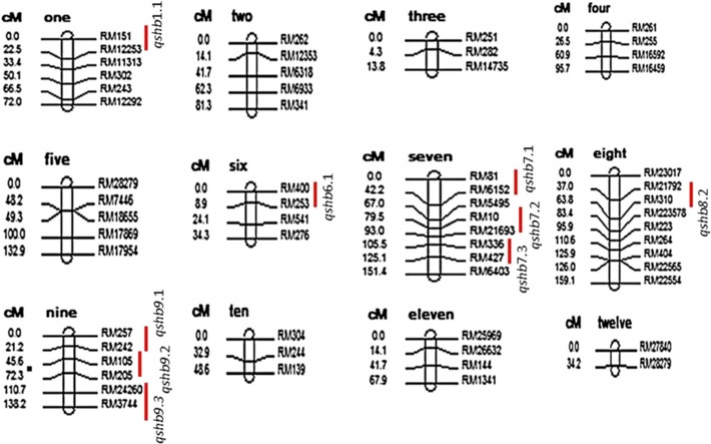
Molecular genetic map of rice along with positions of quantitative trait loci (QTLs) for sheath blight resistance.

Of the 500 markers screened on the parental genotypes (BPT-5204 and ARC10531), 70 SSR markers were found polymorphic. The percent polymorphism between BPT-5204 and ARC10531 was low. Low level of parental polymorphism can be attributed to the narrow genetic variation between the parents as both were indica ecotypes and adapted to grow in the same rice ecosystem. Several line of evidence are in support which also indicate low level of polymorphism between the parents in the intra-sub-specific (Ali et al. 2000; Subashri et al. 2009; Gomez et al. 2010) and even in inter-sub-specific crosses of rice (McCouch et al. 1988; Price and Tomos, 1997). The polymorphic markers were used for genotyping of the F_2:3_ mapping population. Majority of the loci followed Mendelian ratio i.e., 1:2:1 except ten markers. Based on the F_2_ population, a genetic linkage map including 60 markers evenly distributed over all the 12 chromosomes was constructed and used to identify QTLs for rice sheath blight resistance. Using CIM analysis, a total of 9 QTLs were identified on chromosomes 1, 6, 7, 8 and 9 (Table 3). The 3 QTLs were identified on each of the chromosomes 7 and 9. Some of the QTLs identified in the present study have also been reported in previous studies, suggesting the existence of some conserved chromosomal regions linked with ShB resistance (Tan et al. 2005; Liu et al. 2009; Wang et al. 2012; Shiobara et al. 2013; Zuo et al. 2014). However, some new ShB QTLs were also identified on chromosome 1, 7 and 8 (Figure 6) in addition to those earlier reported.

**Table 3:** **QTLs identified for Sheath Blight Resistance by Composite Interval Mapping (CIM)**

**S.no**	**QTLs**	**Chr.**	**Marker**	**Marker Interval**	**LOD**	**%R** ^**2**^	**Additive**	**Dominance**
**1**	*qshb1.1*	1	RM151	RM151-RM12253	10.7	10.99	13.9051	−24.7665
**2**	*qshb6.1*	6	RM400	RM400-RM253	4.43	13.25	−9.0513	2.0605
**3**	*qshb7.1*	7	RM81	RM81-RM6152	8.8	10.52	−5.5132	−6.0402
**4**	*qshb7.2*	7	RM10	RM10-RM21693	6.7	9.72	−3.0300	−2.9706
**5**	*qshb7.3*	7	RM336	RM336-RM427	4.12	21.76	2.8837	−3.0868
**6**	*qshb8.1*	8	RM21792	RM21792-RM310	4.2	10.52	−3.6675	−3.7066
**7**	*qshb9.1*	9	RM257	RM257-RM242	5.9	8.40	−3.1244	−2.1194
**8**	*qshb9.2*	9	RM205	RM205-RM105	7.0	19.81	3.7999	−4.2493
**9**	*qshb9.3*	9	RM24260	RM24260-RM 3744	3.5	12.58	−3.2724	−2.4682

On chromosome 1, the QTLs *qshb1.1* was found linked to the markers RM151 and RM12253 respectively with phenotypic variance of 10.99% and 12.18%, explaining involvement of additive gene action. Earlier workers too mapped ShB resistance to chromosome 1, but at locations different from the presently identified location (Liu et al. 2009; Jia et al. 2012). For ShB QTL on chromosome 6 (*qshb6.1*), the genomic region identified in the present study were same as the ones reported earlier by Zou et al. (2000) and Liu et al*.* (2009), but the peak markers were different. Thus, the markers linked to the ShB resistances QTLs on chromosome 1, 6 and 8 were different and reported for the first time. Likewise the QTL *qshb8.1* was found at almost the same genomic region as that of the QTL reported by Li et al*.* (1995) and Channamallikarjuna et al. (2010).

On chromosome 7, three QTLS *qshb7.1, qshb7.2*, and *qshb7.3* were mapped and phenotypic variance of 21.76% was observed for QTL *qshb7.3* denoting strong additive gene action. Four studies detected QTLs for sheath blight around the chromosomal region of *qshb7.3* (Pan et al. 1999; Zou et al. 2000; Kunihiro et al*.*^2002^ and Liu et al*.*^2009^).

Some of the chromosomal regions containing ShBR QTLs and its associated markers identified in present investigation were also reported earlier by different researchers. However, the genomic locations of these QTLs were observed to be different in different populations. These might be due to the use of different sources of resistance to sheath blight in different studies, varying methodologies of assessing sheath blight resistance, or the use of different marker densities as earlier expressed by Channamallikarjuna et al. (2010).

A QTL region *qshb7.3* identified on chromosome 7 associated with marker RM336 was in agreement with the previously reported result of Channamallikarjuna et al. (2010) with different genomic location. The alleles explained 21.76% of total phenotypic variance in contrast with the report of Channamallikarjuna et al. (2010) depicting only 10.02% for this QTL. This might be due to interaction of favorable alleles in this source (ARC10531) of resistance. The QTL region *qshb7.3* associated with same peak marker RM336 in both the studies represents the stability of the QTL for sheath blight resistance.

The major QTL *qshb9.2* identified on chromosome 9 associated with marker RM205 was earlier reported by Tan et al*.* (2005) in F_2_ population derived from a Lemont/Teqing. The QTL location, associated marker and phenotypic variance identified in their study was quite similar to that observed in our study. The QTL *qshb9.2* identified in this study not only helped in validation of QTL once again, but also provided an alternate source of resistance for introgression of the QTL into any elite variety. This consistent QTL region *qshb-9* for sheath blight resistance on chromosome 9 has been verified by various independent researches (Li et al. 1995; Tan et al. 2005; Pinson et al. 2005; Liu et al*.*^2009^; Yin et al. 2010; Zuo et al. 2014) and in the present study, indicating its authenticity and potentially breeding value in practice. These may be candidate regions to explore in ShB resistance breeding programme. The major ShB-QTLs *qshb7.3*and *qshb9.2* identified in the present study, as a favorable QTL can be readily transferred using MAS into elite cultivars to strengthen their resistance levels. In addition, we have identified other minor ShB QTLs, which can be used to develop new cultivars with improved resistance to this important disease.

Although the present findings are preliminary and QTL were identified under controlled condition, the validation of identified QTLs in other populations with adequate size and number of markers tested across multiple environments would add additional landmark in regards to identify resistance genes against sheath blight.

### Confirmation of linked microsatellite markers associated with sheath blight resistance in rice

**Figure 7:**
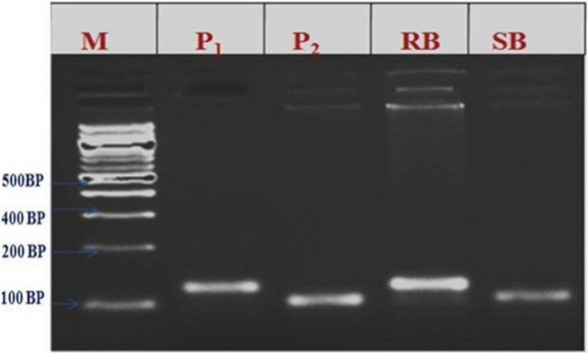
Results of bulk segregant analysis of BC_1_F_2_ population with SSR marker RM 205 M-100 bp ladder; P_1_-ARC10531; P_2_-BPT-5204; RB-Resistance bulk; SB–Susceptible bulk.

**Figure 8:**
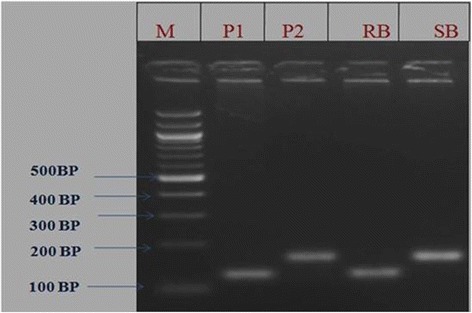
Results of bulk segregant analysis of BC_1_F_2_ population with SSR marker RM 336 M-100 bp ladder; P_1_-ARC10531; P_2_-BPT-5204; RB-Resistance bulk; SB–susceptible bulk.

The two SSR markers (RM336 and RM205) associated with major QTL *qshb7.3* and *qshb9.2* were reconfirmed in BC_1_F_2_ population using bulk segregant analysis approach. The DNA from 10 extreme resistant and 10 extreme susceptible plants were pooled separately and amplified along with both the parents using the same SSR markers RM336 and RM205 (Figures 7 and 8). The alleles were showing co-segregation among the parents ARC10531, BPT-5204 and pooled DNA of resistant bulk and susceptible bulk with SSR markers RM336 and RM205.

Channamallikarjuna *et al.* (2010) utilized an F_2_ population derived from resistant parent Tetep, to confirm the linked marker associated with sheath blight QTL identified in RIL population. They validated the marker RM224 linked to major QTL, *qSBR11-1* identified during their earlier study with RILs of same cross. In the present study, the two microsatellite markers RM336 and RM205 associated with major QTLs, *qshb7.3 and qshb9.2* were reconfirmed in the BC_1_F_2_ population by using BSA approach. The evaluation of stable line phenotypically at the ShB hot spot location to study the stability of the identified major QTL could speed up the process of authenticating markers for phenotypically complex traits viz sheath blight and can be particularly useful for mapping genes whose phenotypes cannot be easily separated into discrete classes.

### *In silico* analysis for presence of defense responsive candidate genes within the identified QTLs

In order to navigate for the presence of candidate defense responsive genes present in the chromosomal regions associated with sheath blight resistance, a search was done *in silico* using rice data base RAP-DB. This *in silico* search was done for the presence of defense responsive genes as well as genes associated with signal transduction of biotic stresses.

The search identified *in silico* a single copy of a hypothetical *β* 1–3 *glucanase* gene using rice database RAP-DB within the identified QTL *qshb9.2* associated with RM205 on chromosome 9. A total of 32 genes were predicted within QTL region near to the marker RM205 on chromosome 9 (Table 4). Functional annotation of predicted genes by blastp revealed 1 defense responsive gene *β* 1–3 *glucanase* like protein present in a single copy within the cluster and it may be responsible for sheath blight resistance in the rice line ARC-10531.

**Table 4:** **List of candidate genes identified in the QTL mapped region** ***qShb 9.2***

**Chr. ID**	**Locus ID**	**Position start – end (bp)**	**Description (RAP-DB annotation)**
**Chr# 9**	Os09g0520550	21076561- 21079025	Hypothetical protein.
	Os09g0522000	21137921- 21138817	Similar to CBF-like protein
	Os09g0525300	21354401- 21357048	Cyclin-like F-box domain containing protein
	Os09g0527900	21478060- 21481626	Similar to Hd1-like protein
	Os09g0528700	21523011- 21524758	Cytochrome P450 family protein
	Os09g0530800	21626098- 21629408	Proteinase inhibitor, propeptide domain containing protein
	Os09g0533200	21770245-21771297	Similar to β-1, 3-glucanase precursor (EC 3.2.1.39).
	Os09g0534600	21844366- 21849310	Similar to Heat shock protein 82
	Os09g0534800	21860615- 21863638	Transcription initiation factor IIB (TFIIB).
	Os09g0535200	21877935-21881669	Conserved Hypothetical protein.
	Os09g0535400	21886226-21887515	Curculin-like (mannose-binding) lectin domain containing protein.
	Os09g0536000	21895656-21900367	Exodeoxyribonuclease III xth family protein.
	Os09g0537700	21987605-21989587	Ribonuclease T2 family protein
	Os09g0538450	22028070-22028968	Hypothetical protein.
	Os09g0540800	22123789-22125783	Eukaryotic transcription factor, DNA-binding domain containing protein
	Os09g0541000	22141777-22143042	Similar to Plasma membrane intrinsic protein (Aquaporin).
	Os09g0541900	22170483-22173850	Similar to 26S proteasome subunit RPN3a.
	Os09g0543900	22282226-22284281	Transferase family protein.
	Os09g0544400	22328257-22346493	Similar to Glutathione S-transferase GST 16 (EC 2.5.1.18).
	Os09g0544900	22369643-22374563	Glucose/ribitol dehydrogenase family protein.
	Os09g0547800	22484690-22486975	Cyclin-like F-box domain containing protein.
	Os09g0549700	22577011-22578446	Ribosomal protein L18P/L5E family protein.
	Os09g0551000	22629301-22632936	Protein kinase domain containing protein.
	Os09g0553100	22743724-22744310	Histone H4.
	Os09g0553600	22762665-22766553	Similar to NADC homolog.
	Os09g0560200	23092024-23093755	Similar to 26S protease regulatory subunit 6B
	Os09g0562400	23166029-23177080	Similar to mutator-like transposase [Oryza sativa (japonica cultivar- group)].
	Os09g0563300	23214566-23218631	Similar to RuBisCO subunit binding-protein alpha subunit, chloroplast precursor (60 kDa chaperonin alpha subunit)
	Os09g0565400	23345149-23348841	Lipoprotein, type 6 family protein
	Os09g0569200	23544922-23548178	Similar to Beta-amylase (EC 3.2.1.2) (1,4-alpha-D-glucan maltohydrolase).
	Os09g0569300	23548388-23551570	Similar to Calmodulin-binding heat-shock protein
	Os09g0569400	23551978-23559137	Beta-lactamase-like domain containing protein

Zuo et al. (2014) reported 12 candidates within the QTL region *qSB-9*^*TQ*^ responsible for transferase family proteins glutathione S-transferase and kinase protein. Silva et al. (2012) identified four candidate genes within the major QTL *qShB9-2,* mapped by Liu *et al*. (2009). In their study, they found the pathogenesis-related (PR) protein glucan endo-1, 3-β-glucosidase (*glucanase*) at two loci on chromosomes 8 and 9. Interestingly in our study also, same kind of gene (β -1, 3 *glucanase* like protein) with single copy spanning 21770245…21771297 bp was identified on chromosome 9 within the QTL *qshb9.2* linked with marker RM205. A wealth of scientific reports (Sridevi et al. 2003; Kalpana et al. 2006) accumulated on plant *β -1,3-glucanases* has enabled us to understand their structure, regulation of expression and the multitude of roles they play directly and indirectly in plants. *β -1, 3- glucanases* are most studied with respect to their expression during pathogen infection and their broad spectrum anti-fungal, antimicrobial activity (Ignacimuthu and Antony 2012). Channamallikarjuna et al. (2010) predicted 11 tandem repeats of chitinase genes near the QTL *qSBR11-1* on chromosome 11 may be responsible for sheath blight resistance in rice line Tetep. Our study revealed, *β-1, 3-glucanase* precursor (EC 3.2.1.39) a PR protein within QTL (*qshb9.2*) on chromosome 9. Hence, pyramiding of these two major QTLs into one background gives the possibility of combined effect of *chitinase* and *β-1, 3-glucanase* may be the feasible strategy to produce resistant cultivar against ShB.

## Conclusion

In present study two SSR markers namely, RM336 and RM205 were found to be closely associated with the major QTLs *qshb7.3* and *qshb9.*2, respectively in the newly identified source ARC10531 and same were confirmed as well in BC_1_F_2_ population by bulk segregant analysis approach. The presence of defense responsive gene *β* 1–3 *glucanase* as well as genes associated with signal transduction of biotic stresses within ShB resistance QTL (*qshb9.*2) may be responsible for sheath blight resistance in rice line ARC10531. Further, the approach of pyramiding QTLs identified in this source with earlier identified diverse QTLs for ShB resistance may help in developing cultivars with enhanced and durable resistance to this disease.

## Additional file

Additional file 1: Table S1. Standard Evaluation System (SES*) scale (0-9) for Sheath Blight disease scoring *IRRI 2002. **Table S2.** List of Polymorphic SSR Markers with their details.
