# Supplementary material for: Identification of QTLs and possible candidate genes conferring sheath blight resistance in rice (Oryza sativa L.)
Source: Springerplus. 2015 Apr 11;4:175. doi: 10.1186/s40064-015-0954-2 (PMC4414854; doi:10.1186/s40064-015-0954-2)
Supplement: Additional file 1: Table S1. — Standard Evaluation System (SES*) scale (0-9) for Sheath Blight disease scoring *IRRI 2002. Table S2. List of Polymorphic SSR Markers with their details. [file 40064_2015_954_MOESM1_ESM.docx]

| **Disease score** | **Reaction** | **Description (based on relative lesion height)** |
| --- | --- | --- |
| 0 | Immune | No infection |
| 1 | Resistant | Vertical spread of lesion up to 20% of plant height |
| 3 | Moderately Resistant | Vertical spread of lesion up to 21-30% of plant height |
| 5 | Moderately Susceptible | Vertical spread of lesion up to 31-45% of plant height |
| 7 | Susceptible | Vertical spread of lesion up to 46-65% of plant height |
| 9 | Highly Susceptible | Vertical spread of lesion up to 66-100% of plant height |

**Table S1 Standard Evaluation System( SES*) scale (0-9)for Sheath Blight disease scoring *IRRI 2002**

| **Sl.No.** | **Marker name** | **Chrom. No.** | **Motif** | **PCR Product Size** | **Annealing Temp.** |
| --- | --- | --- | --- | --- | --- |
| 1 | RM151 | 1 | (TA)23 | 197 | 55 |
| 2 | RM12253 | 1 | (AAT)26 | 351 | 56 |
| 3 | RM11313 | 1 | (TTA)23 | 388 | 55 |
| 4 | RM10615 | 1 | (AGA)31 | 288 | 57 |
| 5 | RM302 | 1 | (GT)30(AT)8 | 156 | 55 |
| 6 | RM243 | 1 | (AT10 | 150 | 56 |
| 7 | RM562 | 2 | (AAG)13 | 126 | 56 |
| 8 | RM12031 | 2 | (AG)48 | 296 | 57 |
| 9 | RM262 | 2 | (CT)16 | 154 | 56 |
| 10 | RM12353 | 2 | (TAT)26 | 452 | 56 |
| 11 | RM6318 | 2 | (CTT)12 | 199 | 56 |
| 12 | RM6933 | 2 | (TTA)22 | 215 | 56 |
| 13 | RM12941 | 2 | (AGAT)12 | 186 | 57 |
| 14 | RM341 | 2 | (CTT)20 | 172 | 56 |
| 15 | RM183 | 2 | (GA)8 | 222 | 55 |
| 16 | RM251 | 3 | (CT)29 | 147 | 57 |
| 17 | RM282 | 3 | (GA)15 | 136 | 56 |
| 18 | RM14735 | 3 | (AT)42 | 295 | 56 |
| 19 | RM 14778 | 3 | (AT)37 | 187 | 57 |
| 20 | RM 14270 | 3 | (AT)26 | 353 | 55 |
| 21 | RM261 | 4 | C9(CT)8 | 125 | 56 |
| 22 | RM255 | 4 | (AGG)5(AG)2-(GA)162 | 144 | 55 |
| 23 | RM16592 | 4 | (ATAC)29 | 393 | 57 |
| 24 | RM16459 | 4 | (AAT)16 | 190 | 55 |
| 25 | RM 17620 | 4 | (AT)34 | 324 | 55 |
| 26 | RM28279 | 5 | (TTGA)8 | 299 | 56 |
| 27 | RM7446 | 5 | (TAAA)22 | 188 | 56 |
| 28 | RM18655 | 5 | (AAG)22 | 200 | 56 |
| 29 | RM17869 | 5 | (CGC)7 | 384 | 55 |
| 30 | RM17954 | 5 | (TTA)26 | 197 | 56 |
| 31 | RM400 | 6 | (ATA)63 | 155 | 55 |
| 32 | RM253 | 6 | (GA)25 | 141 | 55 |
| 33 | RM541 | 6 | (TC)16 | 158 | 55 |
| 34 | RM276 | 6 | (AG)8A3(GA)33 | 149 | 56 |
| 35 | RM81 | 7 | (TCT)10 | 110 | 56 |
| 36 | RM6152 | 7 | (CGC)9 | 206 | 56 |
| 37 | RM5495 | 7 | (TC)24 | 194 | 56 |
| 38 | RM10 | 7 | (GA)15 | 159 | 56 |
| 39 | RM21693 | 7 | (AT)44 | 462 | 56 |
| 40 | RM336 | 7 | (CTT)18 | 154 | 56 |
| 41 | RM6403 | 7 | (GAG)8 | 98 | 56 |
| 42 | RM 20834 | 7 | (AC)43 | 267 | 57 |
| 43 | RM23017 | 8 | (TAA)18 | 133 | 56 |
| 44 | RM21792 | 8 | (AATT)5 | 197 | 50 |
| 45 | RM310 | 8 | (GT)19 | 105 | 55 |
| 46 | RM22578 | 8 | (AT)29 | 498 | 56 |
| 47 | RM223 | 8 | (CT)25 | 155 | 56 |
| 48 | RM264 | 8 | GA)27 | 178 | 56 |
| 49 | RM404 | 8 | (GA)33 | 236 | 56 |
| 50 | RM22565 | 8 | (TGTA)15 | 276 | 56 |
| 51 | RM22554 | 8 | (ATC)13 | 3 | 56 |
| 52 | RM257 | 9 | (CT)24 | 147 | 57 |
| 53 | RM242 | 9 | (CT)26 | 225 | 56 |
| 54 | RM105 | 9 | (CCT)6 | 134 | 56 |
| 55 | RM205 | 9 | (CT)25 | 122 | 56 |
| 56 | RM24260 | 9 | (ATA)31 | 244 | 57 |
| 57 | RM 23741 | 9 | (AAT)28 | 341 | 56 |
| 58 | RM3744 | 9 | (GA)17 | 151 | 57 |
| 59 | RM304 | 10 | (GT)2(AT)10(GT)33 | 160 | 56 |
| 60 | RM244 | 10 | (CT)4(CG)3C(CT)6 | 163 | 56 |
| 61 | RM139 | 10 | (CT)5 | 386 | 57 |
| 62 | RM25969 | 11 | (AAG)18 | 339 | 56 |
| 63 | RM26632 | 11 | (TCTT)9 | 434 | 56 |
| 64 | RM144 | 11 | (ATT)11 | 237 | 56 |
| 65 | RM1341 | 11 | (AG)22 | 183 | 57 |
| 66 | RM27840 | 12 | (TAT)37 | 508 | 56 |
| 67 | RM28279 | 12 | (TTGA)8 | 299 | 55 |
| 68 | RM28781 | 12 | (ACG)7 | 268 | 55 |
| 69 | RM28732 | 12 | (ACG)7 | 181 | 55 |
| 70 | RM28795 | 12 | (AT)15 | 234 | 55 |

**Table S2 List of Polymorphic SSR Markers with their details**
